# Comparison of intraocular lens power calculation in simultaneous and sequential pterygium and cataract surgery

**DOI:** 10.22336/rjo.2021.31

**Published:** 2021

**Authors:** Rajesh Subhash Joshi, Sudhir Sudhakar Pendke, Shobha Marewar

**Affiliations:** * Department of Ophthalmology, Vasantrao Naik Government Medical, College, Maharashtra, India

**Keywords:** pterygium, phacoemulsification, combined cataract surgery and pterygium excision, biometry

## Abstract

**Objective:** To evaluate the effect of pterygium excision on intraocular lens (IOL) power and refraction.

**Methods:** The present study was carried out on patients with combined cataract and pterygium excision (combined group) and pterygium surgery first and cataract surgery after one month (sequential group). Parameters such as mean keratometry (K) values, axial length, IOL power, and corneal astigmatism were compared pre and postoperatively in the combined and sequential groups.

**Results:** 70 eyes of 70 patients were included in the present study. The mean age of the participants in the combined group was 70.46±10.12, whereas that in the sequential group was 68.68±11.22 (p=0.243). The mean horizontal length of the pterygium in the combined group was 2.64±0.17 mm and 2.57±0.17 mm in the sequential group. The mean postoperative K values (p=0.03) and IOL power (p=0.04) in the combined group were significantly higher than the preoperative values. The estimated postoperative refractive error in the combined group was -0.50±1.00 D and 0.25±0.5 D in the sequential group (p=0.04). On the other hand, the postoperative refraction in the sequential group was predictable. Corneal visibility was diminished on the nasal side in almost all the patients in the combined group as compared to the sequential group.

**Conclusion:** The postoperative refraction errors were positively correlated with the length of pterygium in the combined group. The unpredictability of these errors recommends sequential surgery in cases with concurrent pterygium and cataract.

## Introduction

Pterygium is a triangular-shaped conjunctival overgrowth extended to the cornea. It is a common ocular disease associated with ocular irritation and visual impairment and could induce astigmatism. The current standard treatment for this condition is surgical excision, although high recurrence rate is yet an unresolved issue [**1**]. The surgery significantly increases the spherical power of the cornea, while a significant decrease was observed in astigmatism [**2**]. 

Pterygium is a common condition in elderly patients and often accompanied by cataract. The exposure to ultraviolet radiation leads to the co-occurrence of pterygium and cataract, and hence, a high incidence of both conditions is noted in tropical countries. A study from central India demonstrated that the prevalence of pterygium was associated with the exposure to sunlight as well as outdoor activities [**3**]. Nirmalan et al. have shown an increased prevalence of cataract in rural population of India [**4**]. 

Intraocular lenses (IOLs) are artificial medical devices that replace the natural lens of the eye, which has turned opaque and, hence, is removed during cataract surgery. However, the power of these IOLs to be implanted is a major concern in patients undergoing combined cataract and pterygium surgery. The technological advancements in cataract removal have raised the expectations of vision quality. Nonetheless, accurate IOL power is obligatory for improved visual and refractive outcomes. Kamiya et al. assessed the predictability of IOL power calculation after simultaneous pterygium and cataract surgery with IOL implantation in a retrospective analysis and concluded that the myopic shift of refraction postoperatively occurred due to the steepening of cornea [**5**]. Koc et al. studied the effect of the size of pterygium on IOL power in patients who underwent bilateral pterygium surgery without having cataract. The study suggested that IOL power should be 0.50 D smaller than the calculated IOL power when the size of the pterygium is > 2.44 mm [**6**]. 

To the best of our knowledge, no systematic studies have been carried out on the predictability of IOL power in either the combined or sequential cataract and pterygium surgery in a randomized pattern. Thus, the present study aimed to assess the refractive accuracy of the calculated IOL power. 

## Materials and Methods

**Sample size:** To find a 25% difference between the simultaneous and sequential groups with 80% power, 5% significance level, and allowing 10% loss to follow-up, the necessary sample size was determined to be 35 eyes in each group.

**Patient selection and study design:** This study was carried out according to the protocols of the Declaration of Helsinki. The ethical approval was obtained from the medical ethics committee of the hospital. An informed consent was also obtained from all the participants.

This prospective, comparative, and randomized case series included patients with combined cataract and pterygium excision (Combined group) and pterygium surgery first and cataract surgery after one month (Sequential group). The duration of the study was from May 2017 to July 2018. Exclusion criteria were the following: previous history of ocular trauma or surgery, recurrent pterygium, temporal pterygium, combined nasal and temporal pterygium, corneal ectasia, and pterygium < 2.00 mm and > 3 mm. Preoperative assessment included best-corrected visual acuity, slit lamp examination, and keratometry (K) on autorefractor keratometer (Topcon KR-800, Topcon Medical Systems Inc., Oakland, NJ, USA). This keratometer performs K reading in the central 3.2 mm of the cornea. Both flat (K1: angle of flat axis, K2: angle of steep axis) and steep axis measurement was performed. Hand-held tonometer (Perkins, Haag-Streit, UK Ltd., Harlow, UK), retinal evaluation, and A-scan biometry (Axis-II PR Biometer, Quantel Medicals, Cournon-d’Auvergne, France) were used to assess the axial length measurement and to calculate the IOL power. The IOL power was calculated with an A-constant of 118.0 using the SRK II formula.

Simple randomization method was followed by toss method. The heads were assigned to the combined and tails to the sequential group. 

**Surgical technique:**

**Pterygium excision**

All the patients were operated on by a single surgeon. The lignocaine jelly (Xylocaine Jelly 2% AstraZeneca India Ltd.) was instilled in the conjunctival sac 10 min before the surgery. The size of the pterygium was measured with digital calipers from the limbus to the apex. A vertical incision was facilitated over the body of pterygium 2 mm behind the limbus. The head of the pterygium was dissected from the cornea with blunt dissection. The subconjunctival tissue from under the body of the pterygium was removed. The bleeding points were cauterized with wet field cautery. The body of the pterygium was kept retracted on to the bare sclera, and the area underneath was dried with a cotton bud. A free conjunctival autograft was excised from the superior bulbar area. The graft size was obtained by measuring the area of exposed sclera with the calipers. Then the graft was positioned over the bare sclera in the nasal area with limbus-to-limbus orientation for 10 minutes by applying gentle pressure with a sponge. After drying, the redundant margins of the graft were excised with Vannas scissors, and the lid speculum was removed without subsequent suturing. The eye was bandaged for 24 h.

**Cataract surgery**

All the surgeries were performed by the same surgeon. An equivalent of 0.5% proparacaine hydrochloride eye drops were instilled topically two times every 10 min before the surgical procedure. A 20-G side-port incision was created on the appropriate side as required. Then, viscoelastic (2% hydroxypropyl methylcellulose, Appavisc, Appasamy Ocular Devices, Puducherry, India) was injected through the side port using a 23-G blunt tip cannula. Next, a 2.8-mm clear corneal temporal incision was performed. Subsequently, continuous curvilinear capsulorhexis was facilitated using Utrata forceps under viscoelastic conditions. Hydrodissection was performed using balanced salt solution (BSS). Hydrodelineation was carried out in patients with posterior polar cataracts. The nucleus was managed by the direct chop method at the following settings: power 90% (linear), aspiration flow rate 34 cc/ min, and vacuum 350 mmHg. These parameters were not modified in any of the cases until the last fragment was emulsified. A thorough cortical clean-up was accomplished by irrigation and aspiration. The anterior chamber was filled with ophthalmic viscosurgical device single-piece hydrophilic IOL (Acryfold, Appasamy Ocular Devices) with a 6-mm optic diameter, dual haptics, 12.5-mm overall length, biconvex optic design, and square edge design. Subsequently, the anterior chamber was washed to clear the viscoelastic device, and stromal hydration of the side port and the main incision were carried out in the presence of BSS. 

All surgeries were completed without adverse events. Emmetropic power was selected in both groups, and the amount of postoperative ametropic power was noted.

The prediction spherical error was calculated by subtracting the predicted postoperative refraction from the postoperative spherical equivalent at one month. 

**Combined pterygium and cataract surgery**

The procedure was the same as the one described above. 

**Postoperative estimate refractive error**

This parameter was calculated by subtracting the projected postoperative refraction (spherical) from the postoperative refractive error (sphere) at 1-month interval. 

**Statistical analysis:** The data were entered in Excel sheet (Software version 14.1.0 [110310]/ 2011) (Microsoft Corporation, Redmond, WA, USA), and statistical analyses were performed using SPSS version 13.0 (SPSS Inc., Chicago, IL, USA). Chi-square test and t-test were used for categorical and continuous variables, respectively. Values were expressed as mean ± standard deviation (SD) and percentage as appropriate. A *p*-value < 0.05 was considered statistically significant. 

## Results

A total of 70 eyes of 70 patients were included in the present study.

During surgery, the mean age of the participants in the combined group was 70.46 ± 10.12 (range, 60–90) years, whereas that in the sequential group it was 68.68 ± 11.22 (range, 59–90) years (p=0.243). The mean horizontal length of the pterygium in the combined group was 2.64 ± 0.17 (range, 2.10–3) mm and 2.57 ± 0.17 (range, 2.20-3) mm in the sequential group. The pre- and postoperative mean K values, axial length, IOL power, and corneal astigmatism are presented in **Table 1**. 

**Tabel 1:** Evaluation of the parameters pre- and postoperatively

Parameter	Combined group			Sequential group		
	Preoperative	One month postoperative	*p*-value	Preoperative	One month postoperative	*p*-value
Keratometry (D)	44.36 ± 1.83	45.92 ± 0.25	0.03	44.00 ± 1.74	44.06 ± 1.74	0.453
Axial length (mm)	22.50 (±0.55)	22.48 (±0.58)	0.543	23.02 (± 0.85)	22.99 (± 0.86)	0.324
IOL power (D)	21.79 ± 2.36	22.53 ± 2.3	0.04	20.80 ± 2.31	20.70 ± 2.30	0.334
Corneal astigmatism (D)	2.43 ± 2.0	1.25 ± 1.0	0.02	1.12 ± 0.76	1.00 ± 0.67	0.256

**Fig. 1:**
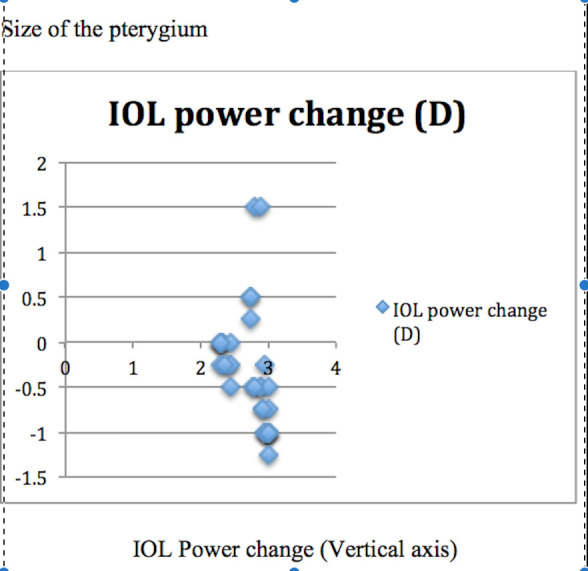
Size of the pterygium and change in the IOL power in the combined group

In the combined group, the mean postoperative K values (p=0.03) and IOL power (p=0.04) were significantly higher than the preoperative values, while no significant change was detected in the axial length. Furthermore, the corneal astigmatism was significantly decreased from 2.43 ± 2.0 preoperatively to 1.25 ± 1.0 postoperatively (p=0.02) in the combined group, while no change was observed in the sequential group. The estimated postoperative refractive error in the combined group was -0.50 ± 1.00 (range, ±0.25-1.5) D and 0.25 ± 0.5 (range, ±0.25-0.75) D in the sequential group, exhibiting a significant difference (p=0.04). Two patients showed postoperative refraction of +1.5 D sphere, and one patient had -1.25 D sphere in the combined group. On the other hand, the postoperative refraction in the sequential group was only predictable. The size of the pterygium and change in the IOL power are depicted in **Fig. 1**,**2**. Unpredictability was noted in the IOL power in the combined group as compared to the sequential group. With increase in the length of pterygium, a high variability was detected in the IOL power. Corneal visibility was diminished on the nasal side in almost all the patients in the combined group as compared to the sequential group.

**Fig. 2:**
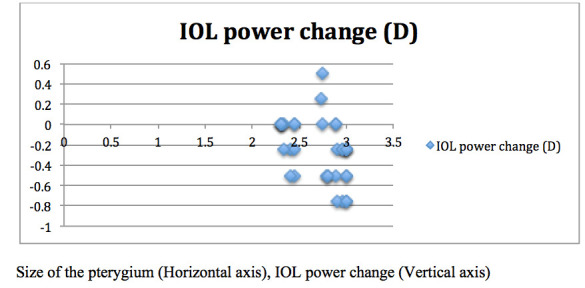
Size of the pterygium and change in the IOL power in the sequential group

## Discussion 

The predictability of refraction after combined cataract and pterygium surgery is challenging due to altered central corneal curvature leading to modified IOL power. It is imperative to decide whether simultaneous cataract and pterygium surgery or sequential will be beneficial in a particular case or not. The present study suggested that sequential pterygium and phacoemulsification surgery with implantation of monofocal IOL is an effective method than the combined technique if the size of the pterygium was > 2.00 mm. Koc et al. and Kamiya et al. showed changes in the horizontal K value on the steeper side, which caused a myopic shift in the postoperative IOL power [**5**,**6**]. To the best of our knowledge, this is the first prospective study that assessed the effect of pterygium excision on IOL power and refraction in the combined and sequential approach. Kamiya et al. studied the predictability of IOL power in patients who underwent simultaneous primary pterygium excision and phacoemulsification with monofocal IOL implantation in a retrospective record review method [**5**]. However, the predictability of IOL power in patients who underwent simultaneous primary pterygium excision and phacoemulsification with monofocal IOL implantation was analyzed in a retrospective study, wherein 82% of the eyes had ±1.0 D of the targeted refraction [**6**]. Furthermore, Koc et al. analyzed the size of pterygium and its effect on biometry in patients with unilateral pterygium [**6**]. Since cataract surgery was not performed, the biometric data were compared to that of the normal eye. 

The current study was carried out on patients with both pterygium and cataract. The size of pterygium was estimated as 2-3 mm in both groups. Kim et al. have shown horizontal K value 3 months post pterygium excision increased by 0.15D in eyes with pterygia smaller than 2 mm and by 0.95 D in eyes with pterygium larger than 2 mm [**7**]. However, Koc et al. considered size of the pterygia more than 2.40 mm in their study on biometric changes after pterygium surgery [**6**]. Nejima et al. have also quantified topographic changes after a large pterygium excision [**8**]. 

The precise way to measure K-reading is the optical biometer. Since it was unavailable in the rural setup, the K-readings were obtained on autorefractor keratometer in the central 3.2 mm of the cornea. It was considered that the horizontal diameter of the cornea had a size of 12-12.5 mm more than > 3 mm of the pterygium, falling in the K-reading measuring zone. Therefore, patients with a pterygium size > 3 mm and combined nasal and pterygium were excluded from this study. 

Topographic changes after recurrent pterygium surgery are unpredictable [**9**,**10**]. Walland et al. have shown astigmatism of 15D in patients having recurrent surgery [**9**]. Thus, patients who had undergone recurrent pterygium surgery were excluded from the study group. 

In the sequential group, an interval of 1 month was maintained between pterygium and cataract surgery. The study by Kim et al. did not reveal any difference in the K value between 1 and 3 months postoperatively in patients with primary pterygium [**7**]. Nejima et al. suggested that the eyes with the advancing edge of the pterygium between one-third of the diameter of cornea and pupillary margin (4 mm) restoration of corneal curvature will recover in 3 months [**8**]. In our study, the size of pterygium was below 4 mm. 

The changes in corneal curvature have been reported previously [**5**,**6**,**11**]. The present study showed there was a significant difference in the postoperative K-value in the combined compared to the sequential group. Similar findings on K-reading were endorsed by Koc, Kamiya, Kim, Errais [**1**,**5**-**7**]. The steepening of the cornea in the combined group led to postoperative changes or errors in the refraction; for example, a large myopic shift occurred with the increase in the length of pterygium. Thus, a significant positive correlation was established between the size of the pterygium and the large myopic shift. Koc et al. advocated that if the horizontal length of the pterygium was < 2.40 mm, only cataract surgery could be performed without causing biometric changes [**6**]. If the pterygium was larger and the patient was planning for combined excision, the IOL power should be at least 0.50 D lesser than the calculated IOL power [**6**]. However, the estimated postoperative refractive error in the combined group was -0.50±1.00 (range, ±0.25-1.5) D. Interestingly, in the present study, one patient exhibited a hypermetropic shift. The unpredictability of the postoperative refractive errors encourages sequential surgery when pterygium and cataract co-exist.

Intraoperative visibility during cataract surgery was also a major concern in the combined approach. The roughness of the surface on the nasal side after pterygium surgery impedes the visualization of the underlying structures, thus necessitating frequent wetting of the corneal surface with BSS or ocular viscoelastic devices. 

With the technological advancements in cataract removal, expectations for better quality of vision are rising. With the development of premium IOL’s and small incisions to remove cataracts, cataract surgery has become a refractive surgery. Patient expects negligible or no postoperative refractive error. Once the corneal curvature stabilizes and shows the significant difference in the corneal curvature one can consider toric IOL for correction of cylindrical power. In the combined pterygium and cataract surgery, toric IOL consideration may not be possible. 

On the other hand, the sequential approach has the disadvantage of increased number of hospital visits and waiting period for cataract surgery. 

## Conclusion

In summary, the present study analyzed the effect of combined and sequential cataract and pterygium surgeries on the IOL power and changes in the refraction in patients with concurrent conditions in the rural regions of India. We found that the IOL power in the combined group was highly unpredictable as compared to that in the sequential group. Notably, the increased length of pterygium caused a high variability in the IOL power. The steepening of the cornea in the combined group caused errors in the refraction, i.e., a large myopic shift occurred with the increase in the length of pterygium. Thus, the unpredictable nature of these changes led to the speculation that sequential surgery is preferred over combined approach in the event of concurrent cataract and pterygium.

**Conflict of Interest statement**

Authors state no conflict of interest.

**Informed Consent and Human and Animal Rights statement**

Informed consent has been obtained from all individuals included in this study.

**Authorization for the use of human subjects**

Ethical approval: The research related to human use complies with all the relevant national regulations, institutional policies, is in accordance with the tenets of the Helsinki Declaration, and has been approved by the institutional review board of the Department of Ophthalmology, Vasantrao Naik Government Medical, College, Maharashtra, India.

**Acknowledgements**

Dr. Avinash Turankar, Associate Professor, Department of Pharmacology, Government Medical College Nagpur, Maharashtra, India for statistical assistance.

**Sources of Funding**

Nil.

**Disclosures**

Nil.

## References

[1] Errais K, Bouden J, Mili-Boussen I, Anane R, Beltaif O, Meddeb-Ouertani A (2008). Effect of pterygium surgery on corneal topography. European Journal of Ophthalmology.

[2] Gulani A, Dastur Y K (1995). Simultaneous pterygium, and cataract surgery. Journal of Postgraduate Medicine.

[3] Nangia V, Jonas JB, Nair D, Saini N, Nangia P, Panda-Jonas S (2013). Prevalence and associated factors for pterygium in rural agrarian central India. The Central India Eye and Medical Study. PLoS One.

[4] Nirmalan PK, Padmavathi A, Thulasiraj RD (2003). Sex inequalities in cataract blindness burden and surgical services in south India. British Journal of Ophthalmology.

[5] Kamiya K, Shimizu K, Iijima K, Shoji N, Kobashi H (2015). Predictability of intraocular lens power calculation after simultaneous pterygium excision and cataract surgery. Medicine.

[6] Koc M, Uzel MM, Aydemir E, Yavrum F, Kosekahya P, Yılmazbaş P (2016). Pterygium size and effect on intraocular lens power calculation. Journal of Cataract and Refractive Surgery.

[7] Kim SW, Park S, Im CY, Seo KY, Kim EK (2014). Prediction of mean corneal power change after pterygium excision. Cornea.

[8] Nejima R, Masuda A, Minami K, Mori Y, Hasegawa Y, Miyata K (2015). Topographic changes after excision surgery of primary pterygia and the effect of pterygium size on topograpic restoration. Eye Contact Lens.

[9] Walland MJ (1994). The effect of recurrent pterygium on corneal topography. Cornea.

[10] Ozgurhan EB, Kara N, Bozkurt E, Gencer B, Yuksel K, Demirok A (2014). Comparison of conjunctival graft thickness after primary and recurrent pterygium surgery: anterior segment optical coherence tomography study. Indian Journal of Ophthalmology.

[11] Tomidokoro A, Miyata K, Sakaguchi Y, Samejima T, Tokunaga T, Oshika T (2000). Effects of pterygium on corneal spherical power and astigmatism. Ophthalmology.

